# Long-term participation 7-8 years after stroke: Experiences of people in working-age

**DOI:** 10.1371/journal.pone.0213447

**Published:** 2019-03-13

**Authors:** Karin Törnbom, Jörgen Lundälv, Katharina S. Sunnerhagen

**Affiliations:** 1 Research group for Rehabilitation Medicine, Section for Clinical Neuroscience, Institute of Neuroscience and Physiology, Sahlgrenska Academy, University of Gothenburg, Gothenburg, Sweden; 2 Centre for Person-Centred Care (GPCC), University of Gothenburg, Gothenburg, Sweden; 3 Department of Social Work, University of Gothenburg, Gothenburg, Sweden; Beth Israel Deaconess Medical Center, UNITED STATES

## Abstract

**Objective:**

To enhance the understanding of long-term participation in working-aged people 7–8 years after stroke.

**Methods:**

This study had a qualitative design, using a thematic analysis methodology. Eleven individuals took part in an in depth interview 7–8 years after a first time stroke. They had received care at the Sahlgrenska University Hospital in Gothenburg, and were recruited as a heterogenic sample with respect to age, gender, stroke severity and subtype.

**Results:**

From the participants’ experiences four themes emerged: “Returning to work after stroke”; “Working life 7–8 years after stroke”; “Social life 7–8 years after stroke”; and “A state of reorientation in life”. Quotes about experienced participation in everyday life were summarized and presented as “Participation after stroke narratives”. Participants chose to emphasize on work- and social life when describing situations of successful participation. Being included in the wider community and having a sense of purpose, when interacting with others, were factors that these narratives had in common. Participants had gradually become accustomed to a somewhat altered life situation. Some consequences after stroke were still considered frustrating in social or work situations. However, the importance of these issues had reduced and were no longer problematized.

**Conclusions:**

Participants felt content with their everyday life in general, which was a principal and positive result of this study. Reaching a stage of acceptance seemed to be a complex and continuous struggle, and an individual approach in long-term rehabilitation would be valuable to support this personal process. More knowledge about what factors that facilitate participation in people of working-age many years after stroke is needed, so that more people can reach a state of positive identity and participation.

## Introduction

Stroke is a leading cause of disability across the developed world, affecting an increasing number of people of working-age [1, 2]. In Sweden, around 25 000 people experience a stroke each year, and 20% are under the age of 65 years [3], placing special demands on rehabilitation and re-integration into society [4].

After stroke, community re-integration and participation are goals in many policy documents, but also complex processes that require individual strength, social support, help from health care professionals as well as a compassionate general public [5–7]. Physical and cognitive deficits, or emotional challenges like fatigue are common and can be seen as difficult barriers to overcome [8–10]. In a long-term perspective, these impairments may still have an impact on participation in everyday life, even for people with mild stroke who are under 65’ [10, 11].

An individual´s experience of involvement in an activity is central to the concept of participation [12]. The following definition of participation has been used for this study; “participation occurs at the intersection of what a person can do, wants to do, has the opportunity to do, and is not prevented from doing in the world where the person seeks to participate” [12]. It is a subjective and individual experience that needs to be understood from a personal perspective. Participation is also formed in a social context and influenced by numerous, partly synergetic factors, which makes it hard to fully comprehend without taking a qualitative approach [5, 11].

Although the literature on stroke rehabilitation is large, not many papers study long-term consequences and experiences held by people of working-age [13, 14]. Many individuals of working-age will live for a long time after stroke, with major responsibilities during a demanding phase of life [4]. In addition, there is an increasing incidence of stroke in younger people in the Western world [15]. Therefore, more knowledge about long-term participation based on subjective experiences held by individuals with stroke, is needed. This can help finding out how effective and sustainable interventions aiming to meet the specific needs of this group many years post-stroke can be developed [16].

In this article, we seek to understand how participation was experienced in everyday life, by individuals of working-age, 7–8 years after stroke. The aim was to obtain a deeper understanding of how participants coped in everyday life, and how they reflected upon their own participation.

## Materials and methods

This study adheres to the consolidated criteria for reporting qualitative research (COREQ) guidelines [17]. It is based on in-depth interviews about views of participation in everyday life among individuals 7–8 years after a first time stroke.

The research team consisted of a female doctoral student, trained as a social worker (KT), a male University Lector and PhD, trained as a social worker (JL) and a female Professor, MD and PhD (KSS). All authors have prior experience and training in the field of qualitative research.

### Sampling and participants

Participants were recruited from the extended Stroke Arm Longitudinal Study at the University of Gothenburg (SALGOT-extended) [18, 19]. They were patients with stroke at Sahlgrenska University hospital between February 4, 2009 and December 2, 2010. All interviews were conducted in 2017, thus 7–8 years had passed since participants had their stroke.

Inclusion criteria were: first time stroke according to International Classification of Diseases (ICD) codes 161 intracerebral haemorrhage, or 163 ischemic stroke, living within 35 km of the hospital, working-age (18–65 years), sufficient verbal and memory ability to understand the questions and being able to take part in an interview.

To gain broad information about the research questions, eligible participants were purposively selected with varying age, gender, stroke severity, and subtype. The list of eligible participants now included individuals with a variety in personal characteristics and were at this stage randomly selected to be contacted by phone. They were asked if they wanted to take part in an interview about their lives after stroke. When 15 people had confirmed an interest in the study they were sent a letter with information about the purpose of the study, a consent form and brief information about the research team as well as the experience of the interviewer (KT). A week later, a researcher rang back to invite participants for an in-depth interview. Subsequently, four people withdrew their interest, and a total of 11 participants were included in the study. Data retrieved from medical charts and information about occupation and living situation from the interviews are presented in Table 1.

**Table 1:** Characteristics of study participants, n (11).

Age, mean, SD (min-max)	48 years, 10 (32–61)
***Gender***	
Women	4
Men	7
***Living situation***	
Alone	3
Cohabiting	3
Married	5
***Mobility***	
No mobility aid	11
***Stroke subtype***	
Ischemic stroke	8
Intracerebral hemorrhage	3
***Lesion side***	
Left	4
Right	4
Bilateral	3
***NIHSS at admission***	
Very mild (0–2)	7
Mild (3–4)	2
Moderate (5–15)	0
Severe (16–42)	2
***mRS at discharge***	
Functionally independent (0–2)	6
Functionally dependent (3–6)	5
***Vocational status***	
Working full-time	8
Working part-time	1
Unemployed	1
Parental leave	1
***Occupations***	Auto mechanic Taxi driver Process leader of a company Self-employed entrepreneur, building construction Textile designer Pastor Travel organizer Medical secretary Midwife Civil engineer

Abbreviations: mRS, modified Rankin Scale. NIHSS, National Institutes of Health Stroke Scale

All participants were independent in activities of daily living and lived in ordinary housing without home service.

### Data collection

The interview guide and potential areas and questions to include were discussed and developed in cooperation with a patient representative from the Swedish Stroke Association. When raising issues and research priorities held by disabled people’s organizations, the research agenda of academics can be more relevant thanks to their contribution of unique insights and understanding [20]. The patient representative stressed that people after stroke might find it difficult to cope with too much social input at the same time. In collaboration, questions about possible differences concerning social life, before and after stroke were added, as well as a general question about how social life was perceived. The question that people might think differently about their lives after stroke was also formulated together with the patient representative. After this, the interview guide was pilot tested on two individuals (a professor physician and a nurse assistant) with long-term stroke who agreed to participate as partners in research in the current study. One of the partners in research had had a stroke in the right hemisphere, with typical residual physical symptoms and the other partner had gotten a stroke in the left hemisphere with aphasia. However, none of the participants included in this study were diagnosed with aphasia. Further revisions were made after input from the partners in research and subsequently a final version of the interview guide was developed. One of the partners in research was interested in conditions at work after stroke and how work tasks were perceived after stroke, hence questions about that were added. The other partner was interested in health issues after stroke, and the three last questions in the “*Health*” section were constructed with input from her.

All interviews (n = 11) were individual and conducted face-to-face by the first author (KT), Master in Social Sciences (MSSc), trained and experienced in interview methods. The interviews began with opening questions concerning demographics and living situation. After that, questions were open-ended and focused on areas relevant to the research questions: how participation was experienced in different life situations 7–8 years after stroke, and how participants’ coped in everyday life.

Follow-up questions were flexible depending on participants’ answers, and therefore the interviews were open with rich content. The interviewer encouraged participants to speak freely, which allowed additional thoughts and topics to emerge.

As data collection carried on, a few questions were added in order to target findings that needed further attention. The interview guide can be found in its complete form as supporting information in both English and Swedish.

Participants seemed enthusiastic and/or interested in the topics that they spoke about, indicating that areas covered during the interview guide were perceived as interesting. Clarifications were used at all times when the interviewer was uncertain about the meaning of what was being discussed. For example; “Did I understand you correctly, you think that being able to work is a high priority in your life?”

All interviews (n = 11) were held from December 2016 to February 2017, and took place in a quiet room at the Sahlgrenska Academy. Upon request, two participants were interviewed in their homes. Interviews lasted for between 36–65 minutes and were recorded and transcribed verbatim. After each interview field notes with spontaneous reflections about the interview were taken. After 11 interviews, no more emerging findings or meaningful information about the research questions were obtained. Therefore data was considered saturated and no additional interview person was included.

### Ethical considerations

The study followed the Helsinki declaration and was approved by the Regional Ethics Committee in Gothenburg (EPN) (Dnr: 225–08) with an additional application, Dnr: T801-10. Participants gave informed written and verbal consent prior to the interview. Ethical problems or dilemma were not identified during the course of this study [21].

### Analysis

Interview data was transcribed verbatim, then imported and sorted in the qualitative data analysis programme NVivo [22]. Data was analyzed with inductive thematic analysis, following established guidelines as described by Braun and Clarke [23]. Thematic analysis is a commonly used method for identifying, analyzing and reporting themes within data. Often it goes further than this, and interprets various aspects of the research topic [24].

To become familiarized with the data, all interviews were read and re-read, enabling patterns and initial meanings to emerge inductively. All codes that could be potentially interesting for the research questions were marked, using color codes. Mind-maps were used to get an overview of all codes, and to see how they could be merged into themes. This approach complemented the analysis in NVivo, and was used to visualize and simplify the process.

The next phase involved refining themes and subthemes. In this phase, some subthemes were collated into one, and minor themes, for example “leisure activities” and “views about hospital care” were removed due to inconclusiveness. Themes considered meaningful to the research questions were kept. Focus continuously moved from the whole to parts of the text to ensure validity of the themes in relation to the entire data set. This process of refining themes was carried out in dialogue between the first (KT) and second (JL) authors until themes seemed to represent the dataset as a whole and consensus was reached. Internal homogeneity and external heterogeneity were considered in this phase [25]. The contents of each theme were carefully re-read, as well as each theme in relation to the others, to ensure they were not overlapping [23]. In the end, analysis and results were discussed again, between all three authors, ensuring validity of the themes in relation to the data set. A part of the coding process is illustrated in Table 2.

**Table 2:** Examples from the coding tree.

Data extract	Code	Theme
“It’s hard when I have a customer and I can’t remember where he’s supposed to go. I have gotten used to using the GPS when I drive, so I don’t forget. That’s why I use it, it’s less of a problem then.”	Understanding limitations at work after stroke and adapting a new strategy	Returning to work after stroke
“When you just don’t have the energy for people sometimes…after a long day filled with meetings, then it’s really nice to just be by yourself.”	Experiencing a need to limit the amount of social interactions	Social life 7–8 years after stroke
“Yes, life is more worth living now after my stroke, you think about what has happened, that you’re not dead…I’m not 100% better, but how I am is good/okay.”	Valuing life more after stroke	A state of reorientation in life

In the interviews, participants were asked to describe at least one situation in everyday life when they felt they were successfully participating. As the answers to this question functioned as a complete theme to begin with, it was defined beforehand and thus deductively analyzed and summarized. This theme was labelled “Participation after stroke narratives” and is presented as an introduction in the results section.

## Results

**Fig 1:**
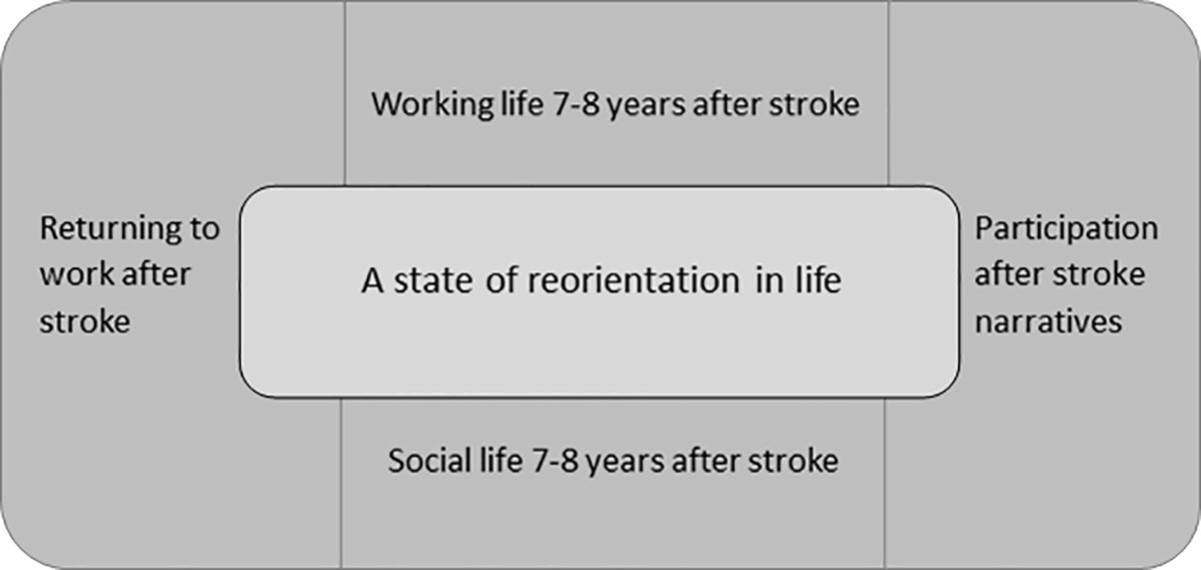
Themes that developed through the analysis. **Long-term participation after stroke**.

Participants’ characteristics and demographics are shown in Table 1. Through the inductive thematic analysis used, four principal themes emerged: 1) *Returning to work after stroke*, 2) *Working life 7–8 years after stroke*, 3) *Social life 7–8 years after stroke*, *4) A state of reorientation in life*. The theme “*Participation after stroke narratives*” (that was analyzed deductively) is presented as an introduction to give an overall picture and better understanding of the results. Fig 1 is an illustration of all themes that are presented.

### Participation after stroke narratives

When participation was described in everyday life, these situations were described as engaging and meaningful. Being included in the wider community and having a sense of purpose, predominantly when interacting with others, were factors that these narratives had in common. Being involved in social situations was described as an important part of enjoying and achieving a sense of meaning in life. Participants also wanted to help others, and to be seen and heard by others.

Some participants highlighted outdoor experiences with beautiful scenery, birds singing or walks in nature when describing involvement in life after stroke. Taking part in quiet and calm activities together with friends or their immediate family were stated to be of higher importance now than before stroke.

When participation was described in a work-related context, participants wanted to play an active role in solving problems and being goal orientated together with their colleagues. Participants also wanted to feel that their skills and competence were appreciated and asked for at work.

*”It is important for me to contribute with something that can be good for me*, *and for others*. *Knowing that I can make a change*! *And that I can receive feedback with good intentions*, *that makes me wanting to participate… Independence and freedom of choice are important as well*.*”* (a 50 year old woman)

### Returning to work after stroke

Handling post-stroke consequences at work was described as a process where participants first had to understand their post-stroke limitations and how these had an impact on their actual work situation. Then, they needed to communicate these problems and in cooperation with people at work find new ways to manage their work tasks, or working hours. If the changes they needed couldn’t be made or weren’t feasible, participants had to change workplaces or work tasks. This was described as emotionally challenging and sometimes accompanied by feelings of guilt or inferiority.

*“Then it’s also that I have to realize*, *mostly for myself…that I can’t process information the same way anymore*. *And that’s taken a very long time for me to accept and understand*. *So it’s an ongoing process for me to tell others when I’m feeling tired*: *“I’m very tired now and this will have to wait until tomorrow*.*”* (a 35 year old woman)

Participants described understanding and supportive leadership as highly important for regaining trust in their own working capacity. Especially when coming back after sick leave, support from the employer had been significantly important in the process of adjusting to new working conditions after stroke.

*“I’m not scared anymore*, *either to say stop*, *this is too much…Or having understanding bosses has played a very big role*. *They have built up my self-confidence*, *they have given me what I need*. *Finding people who believe in you and validate you is incredibly important*.*”* (a 51 year old man)

After stroke, some participants had to change jobs and try multiple workplaces before finding something that suited them. This could be because, for example, they could no longer manage lengthy meetings, too much stress, or working night shift. At some workplaces employers had not been willing to make changes that were needed, and therefore the person with stroke could not return to work after stroke.

### Working life 7–8 years after stroke

At time of the interview, participants said that working was a very important part of their lives. Being somewhere where they felt valuable was expressed as significant for the feeling of participation, and for their self-esteem. When participation at work was described, the opportunity to make personal choices about working procedures and to have control over how to solve problems were important.

*“Independence and opportunity to choose are important*. *Self-determination*. *I can decide for myself how I’m going to organize my day*. *Being able to be creative*, *there isn’t just one solution*, *I get to find my own solutions*. *So I’ve been able to see a positive effect from the stroke; that sometimes I start to think in a completely different way*. *I can think a little outside the box*, *and I think that’s an obvious advantage*.*”* (a 50 year old woman)

Suddenly becoming very tired or unfocused and unable to process information were common everyday consequences after stroke. These problems were handled by taking small breaks, or napping, whenever this was accepted at the workplace. Some stressed that they needed flexible working conditions that enabled working from home or working with breaks. Another strategy was to rest or sleep before or after work.

*” Yeah*, *if I know that I have long board meetings*, *I usually think that it is good if I sleep now*, *because then I’m better rested*. *Or if I’m going to work at festivals and stuff*, *or late nights*. *Then it’s good if I sleep beforehand*.*”* (a 48 year old woman)

An awareness of mind and body signals for overtiredness, and to stress less were common everyday strategies for a successful working life. Participants had learnt that if they chose to ignore such signals, the tiredness would immediately take over, and some said they would also experience irrational emotions, like fear or sadness.

*” The hardest bit is that there isn’t any slow decline for when you get tired*, *but rather*, *you get tired and it’s like a door slamming shut immediately*. *And I do that almost every day at work*, *I just fall asleep for little tiny moments*.*”* (a 32 year old woman)

Another reason for being more careful and taking it easier at work was to avoid recurrent stroke. Some participants had thought about reducing their working hours or changing from night- to dayshifts, but such decisions also depended on their economic situation.

Participants who experienced a deteriorating working memory used notes to avoid forgetting tasks, however, this was not described as a problem. In some situations, having problems finding the right words were experienced as frustrating or embarrassing. Common strategies to handle such situations were to use another word or to wait for the right one to pop up. Participants stressed that these impairments got worse when they felt stressed or had a lack of sleep. Some also expressed uncertainty about whether their abilities had changed after stroke or not. For example, they were not sure if their memory had been better before stroke or if they were just more tired now.

*“That’s a good question…I don’t know* (if having become more tired or not). *I take it much easier now*. *I had a high pace before*. *Both at work and in my free time*.*”* (a 43 year old man)

*“I’ve always had a terrible memory*, *so there’s no real difference now…”* (a 56 year old man)

The view of their professional role and approach to work had also changed 7–8 years after stroke. Several expressed that they now valued personal growth and enjoying being at work more than pursuing a career.

*” I was very career oriented before*, *I wanted to chase after jobs*. *My career for me has developed towards the things I am good at*, *rather than chasing after jobs*. *What I was like before probably wasn’t that good*, *I would go into stress cycles with lots of work tasks…”* (a 51 year old man)

Most participants said they were content with a more relaxed working life, but some felt ambiguous about this;

*” I’m not always the best at taking it easy*. *There’s a discrepancy between what I want to do*, *and what I can actually do*, *that I haven’t really wanted to admit to*. *Because I was often the one who went at it at 200% and then was forced suddenly to back off to 50%*.*”* (a 32 year old woman)

### Social life 7–8 years after stroke

Participants explained that they prioritized spending time with close family and friends to a higher extent 7–8 years after stroke. As life was considered more vulnerable now, significant relationships were valued to a higher degree. Some preferred to socialize with fewer people at the same time and tried not to engage in too many social situations. They put their social energy on substantial, close relationships rather than on strangers or casual acquaintances.

Several chose to describe an example of social participation that was about sharing experiences of difficulties in life. Feelings of being involved emerged from actively listening and talking about each other’s experiences.

*“When I’ve had the opportunity to meet others with their problems*, *then I feel like we have something in common… that this won’t affect us but rather we are looking to the future instead*. *And I do that because I get pushed to*, *and I maybe give others a push to be more positive*.*”* (a 58 year old woman)

Participants stated that they appreciated to be alone at times. In order to rest from social interactions, participants engaged in calm activities like reading a book, listening to music or walking their dog. Some participants also needed to rest before taking part in social activities that they wanted to participate in.

*“This is a residual symptom from the stroke*, *that you sometimes just don’t have the energy for others…*. *I socialize with people when I want to*, *but I don’t have the constant need to be around others*. *I can actually think “God*, *how lovely*, *no-one to talk to*, *hooray*!*”* (a 48 year old woman)

Commonly expressed reasons for being less socially active were; feelings of tiredness or fatigue, difficulties focusing on more than one conversation at the time, or too many distractions.

Participants described that their experienced participation was enhanced when they didn’t feel overloaded by input from their surroundings. Too much noise, colors, or people could interfere with their sense of being in control, for example.

*“I still go out and do things*, *but when I hang out with people*, *and most often when I’m a little tired*, *I have a really hard time in being able to distinguish what people are saying… But I have now pretty much accepted it and I’m like yeah yeah*, *this is kinda nice*, *so sometimes I just switch off and let it be*.*”* (a 35 year old woman)

Participants stated that people generally didn’t seem to understand the concept of fatigue, and because of that, they sometimes found it hard to withdraw from social situations when they needed to.

*“They can understand stuff they can see*. *E*.*g*. *your foot is affected if it doesn’t quite keep up when you walk*. *But mental fatigue is something people don’t get*. *“Why are you so tired*, *why don’t you want to go out with us*?*” It’s not that I don’t want to*, *but it’s my fatigue*, *I really can’t go*.*”* (a 50 year old woman)

Some participants said that helping others had become more meaningful after stroke and that they thought of themselves as more compassionate now. There were also those who felt more emotional after stroke, they cried or laughed more easily, and this was considered as something positive.

### A state of reorientation in life

Participants had, 7–8 years after stroke, reached a state of acceptance in everyday life. They were now used to and comfortable with how their daily routines had changed. It was explained that, over the years, they had found strategies to address consequences after stroke in daily life and that they felt well accustomed to their new way of living.

Several mentioned that through consultations with a psychologist they had been taught how to relate to, for example tiredness or illogical emotions, in a way that made situations easier to handle.

Most participants said that they valued life differently after stroke. Despite not being fully recovered, participants expressed a gratitude for being alive and having coped so well. As a result of this insight some had changed priorities in life. They reflected more about what to engage in and how to prioritize their time and energy.

*” You should be quite humble about life*, *because it can disappear very fast…Some pass away*, *some become disabled for the rest of their life*.. *and I just feel wow*! *So I’m am incredibly thankful that I have got through as well as I have*, *yeah*?*”* (a 43 year old man)

*“I think that it’s impossible to not gain perspective on life*. *Like*, *in the first instance*, *that you’re mortal*! *And that things can go very very fast… I have more empathy now*. *Because there is no other way to understand it until you are sitting there yourself*.*”* (a 32 year old woman)

Participantss were thankful for the new insights they had gained through the process of dealing with life after stroke. Some explained that their personality had gradually changed for the better, and that they thought of themselves as calmer, more thoughtful, or more caring. Reaching acceptance about the lack of control one has over life was described as a part of this process.

*” Yeah*, *I’m calmer now* … *I am a little more thoughtful*, *much more objective*. *It’s like I’ve got access to other sides of myself*. *Focused on solutions*. *I wouldn’t want to be without the experiences I’ve had*, *but the _way_ I got them*, *I could do without*!*”* (a 57 year old man)

As a result of feeling more vulnerable, participants stated that they took better care of themselves, both physically and emotionally, after stroke. They managed stress more effectively. They tried to eat and sleep better and some had cut down on cigarettes and drinking. Because of these lifestyle changes, some said that they, 7–8 years after stroke, were healthier compared to before stroke.

Participants explained that they felt too tired after work to engage in physical exercise. Lack of motivation was also mentioned as a barrier to be physically active, and only one person had found a form of training that they enjoyed.

*” No*, *when you get home at six or seven at night during the week*, *you don’t have much time for working out*. *You shower… Then you are tired again*, *you’re always on the go at work…”* (a 37 year old man)

## Discussion

Participation in everyday life is a complex area to investigate [7] with subjective aspects that need to be taken into account when they are described and evaluated. Personality, preferences, environmental factors, and personal health conditions are examples of important areas that can impact on how a person describes their participation [26]. However, our results showed that in spite of differences, obvious similarities were seen in participants’ reflections about participation in the activities of their choice.

In line with previous findings [5, 7, 27], our results showed that participating in self-selected activities enhanced feelings of belonging, purpose, personal identity, and confidence post stroke. These feelings along with the ability to help others, using one’s competencies to solve problems and to be seen and heard by others were described in the narratives about participation. Spontaneously, participants often chose a social or work-related situation when describing their participation. Similarly to a previous interview study [5], having the ability to choose and decide about activities made participants feel that they were in control, and thus they found it easier to cope in everyday life.

Previous research [28] has suggested that loss of meaningful life roles were related to decreased confidence and of “being a lesser person”. In the present study, personal expectations of one’s professional role had changed, but participants were no longer frustrated or depressed about having somewhat different career goals [27].

In our study, cognitive impairments and fatigue related to stroke were the most commonly expressed reasons for altering daily routines by adapting to accompanying symptoms. Through these adaptations participants explained that their lives now worked well, but nevertheless, participation in daily life was considered limited to some extent.

Although long-term follow-ups about post stroke fatigue are few, it seems that there can be long lasting impairment even after a mild stroke [29–31]. Similarly to another study [32] this study found that cognition and language problems were closely related to fatigue, which further impacted on work and social situations. It was clear that participants had to choose what activities to spend their energy on, and that being able to work full-time was prioritized before social and leisure activities. Social relations were fewer but closer now than they had been before stroke, which was consistent with previous research [6, 10].

In the present study, the majority of participants worked full-time (see Table 1). Work contributed to increased self-esteem, and their job was a meaningful part of their identity [33–35]. In the process of getting back to work, support from employers and well-functioning communication about how work conditions could be adjusted had been highly important. In some cases there was a lack of support, which had made participants feel lonely and vulnerable in the returning to work process. These findings strengthen previous quantitative research [34, 36] which found an association between the level of support and return to work after stroke. Rehabilitation professionals could possibly have a role in the returning to work process, by mediating between workers and employers, and provide information about hidden symptoms since there seem to be a general lack of understanding about them [33, 37].

In agreement with previous research [8, 38], saving energy by incorporating rest during the day was the most commonly used strategy for coping with fatigue. In addition, our findings suggested that adaptations to embody a healthier lifestyle had been made to cope with fatigue. Participants tried to sleep better, drink and smoke less, and to avoid stress at work. However, taking up physical exercise was only described as a part of this adaptation by one person.

It is well known that physical activity levels for individuals after stroke do not reach recommended levels [39]. In the present study participants explained that they had no time to exercise, they felt too tired after work or were not sufficiently motivated. Lack of motivation can result from not valuing an activity because it is not believed to lead to a desired outcome [11]. Additional barriers that may contribute to inactivity are: neurological deficits, cognitive impairments, and environmental factors [40, 41]. Previous research within this field [42–44] suggested a need to adapt long-term perspectives to increase levels of physical exercise after stroke. This is important to prevent a recurrent stroke or another cardiovascular event, but also to improve health and quality of life in individuals after stroke more generally [42].

All participants had transitioned to a stage of reorientation in life 7–8 years after stroke. They had come to terms with and adapted to a changed life situation, thus they reported a quite favorable participation at this stage. This positive result might partly have to do with the younger age of our study group < 65, because a younger age after stroke has been shown [45] to be of great importance for successful participation. In two recent interview studies [16, 26] it was found that in a long-term perspective, stroke was no longer an issue, but had been integrated as a new normality, and other life events were more significant for the experience of participation. In contrast, another interview study [46] which included participants who were 11–13 years after stroke, concluded that only two out of eleven study participants had successfully adapted to their lives after stroke.

In the present study, emphasizing positive aspects of life was both an active strategy to cope, and something that had developed from a genuine gratitude for having survived and managed a serious disease [37, 47, 48]. Participants felt that they wanted to take good care of their lives, and to spend it in a way they considered meaningful. After having accepted a somewhat different way of living, participants mostly experienced well-being in life, even though it had been a long process getting to this stage. In line with previous findings [49] this process of learning about one’s limitations and through trial and error, finding new ways to cope was described as a learning experience. Being aware of personal limitations and reconstructing preferred activities through daily living in a successful way can be referred to as self-management [49]. In a previous interview study post-stroke [50] participants were unfamiliar with the term self-management, but understood it as; “doing things for yourself” or “taking care of yourself”. In addition to these aspects, participants in the current study wanted to prioritize their closest family and friends, before material things or pursuing a career.

A strength of this study was the purposive sampling procedure that aimed for different perspectives to emerge and for variation in data. This, together with the open questioning, made it possible to obtain a deeper understanding of the research questions. A majority of the participants in this study had had a less severe stroke, which is more common in this age group, i.e. people of younger age [51]. The patient perspective was integrated as the interview guide was developed in cooperation with a patient representative from the Swedish Stroke Association. In addition, pilot interviews were performed to develop and improve the interview guide. It is important to facilitate the participation of people living with disabilities in research [20].

A limitation of this study was that questions about the past were asked. It can be difficult to recall memories about what happened several years ago, even if this issue was not expressed by participants. The study group was of working-age, and results might not be representative for older people a long time after stroke. An older population might struggle with issues related to ageing, and participation often decreases with age [52]. Because experiences of participation are sensitive to context and formed in a specific cultural setting, transferability of results to other countries than Sweden needs consideration.

## Conclusions

The results show that participants had learned about how stroke impacted their lives and through trial and error they found ways to adapt in order to experience more successful participation. However, it is still not known exactly what aspects are important for facilitating the process of managing a successful participation. More knowledge about coping strategies that improves participation in activities many years after stroke is needed, so that more people can reach a state of positive identity and participation.

In addition, as participants were not involved in physical exercise to a greater extent, sustainable interventions to promote and encourage people to be more physically active a long time after stroke should be explored.

As long-term participation after stroke seems to be a complex and continuous personal process, an individualized approach in rehabilitation would be valuable. Professional support and encouragement could be needed in the process of adapting one’s behavior and trying to stay positive through challenging life alterations. Support-groups could also be an effective long-term intervention in the workplace to help people in the process of coping with challenges.

## Supporting information

S1 Appendix Interview guide in English. (DOCX)

S2 Appendix Interview guide in Swedish. (DOCX)
