# Supplementary material for: Long-term participation 7-8 years after stroke: Experiences of people in working-age
Source: PLoS One. 2019 Mar 13;14(3):e0213447. doi: 10.1371/journal.pone.0213447 (PMC6415844; doi:10.1371/journal.pone.0213447)
Supplement: S1 Appendix — (DOCX) [file pone.0213447.s001.docx]

**Interview guide**

The interviewer (Karin Törnbom) begins by describing what the conversation is going to be about: “Participation in day to day life, 7-8 years after a stroke”. The interviewer encourages everyone to speak freely, and not to rush their answers.

Different follow-up questions have been used throughout to know more about what the person being interviewed wanted to bring up.

*Familial relationships and social life information*

Do you live live alone? With someone else?

Are there people you meet often and spend time with?

Can you tell me a little about what you usually do together?

Are there other places where you meet people socially?

Is there any difference between how you socialize now versus before the stroke (in what way)?

Do you think your social life is the way you want it to be? (both in how much, and what you actually do when you socialize with other people). If not, is it something that you miss?

*Employment/work (housework)*

Are you gainfully employed, or have any unpaid work, for example do you volunteer anywhere?

Can you describe what it was like to come back to work? (how did it go?)

What did you find to be important when you returned to work?

Are you still working at the same place today?

Could you tell me a little bit about your work?

What do you think about your specific work tasks/commitments?

What does your workload look like compared to your capacity to work?

Is there anything that makes work more difficult for you? (e.g. bodily difficulties, memory problems, fatigue, accessibility).

Would you say that work is important or meaningful to your life?

*Health (problems associated with your stroke)*

Do you have any problems with your health now, after your stroke? It could be something physical or if you are mentally unwell.

Do you go to any kind of rehabilitation?

Do you work out? Is that something you’d want to do?

Do you think you have received the help you need from the hospital/local care clinic with regards to your wellbeing?

Does your health affect your ability to find joy in life? (both physical and mental health)

Do you feel like you are sometimes limited by your health when you are going to do something? (both physical and mental health)

Do you have any tricks or strategies for being able to do what you want anyway? (to overcome physical or mental limitations/difficulties)

Do you have access to support and help from someone close to you with regards to how you are feeling? (both mental and physical health)

*Hobbies (leisure and culture)*

Do you usually do something you particularly like in your free time? (can you tell me a little about it)

Are you able to perform activities (e.g. hobbies or cultural practices) that you like to the extent that you want to?

(If not) What would you say holds you back from being able to perform the activities you like?

*Do you think differently about your life now after stroke?*

Follow up questions (various)

*Has it changed over time, i.e. how do you think about your participation after your stroke?*

Follow up questions (various)

**Ask the participant to define: What does participation mean to you? Can you describe a day to day situation in which you feel as though you are participating?**
