# Supplementary material for: Long-term participation 7-8 years after stroke: Experiences of people in working-age
Source: PLoS One. 2019 Mar 13;14(3):e0213447. doi: 10.1371/journal.pone.0213447 (PMC6415844; doi:10.1371/journal.pone.0213447)
Supplement: S2 Appendix — (DOCX) [file pone.0213447.s002.docx]

# **Intervju guide**

Intervjuaren (Karin Törnbom) inleder med att tala om vad samtalet kommer att handla om: ”Delaktighet i vardagslivet, 7-8 år efter stroke”. Intervjupersonen uppmuntras till att tala fritt, och inte stressa fram svaren.

Genomgående har olika följdfrågor använts för att få veta mer om det som intervjupersonen själv valde att lyfta fram.

*Fakta om familjerelationer och socialt liv*

Bor du ensam eller tillsammans med någon?

Finns det personer som du träffar och umgås med ofta?

Kan du berätta lite om vad ni brukar göra tillsammans?

Deltar du i andra sammanhang där du träffar personer socialt?

Är det någon skillnad i hur du umgås jämfört med innan stroken (på vilket sätt)?

Tycker du att ditt sociala liv ser ut som du önskar? (i mängd och innehåll att du umgås med människor som du önskar) om Nej: är det något som du saknar?

*Sysselsättning/arbete (hemarbete)*

Ägnar du dig åt något betalt, förvärvsarbete, eller obetalt arbete såsom volontärarbete eller ideellt arbete?

Kan du beskriva hur det var att komma tillbaka till jobbet? (hur gick det?)

Vad upplevdes som viktigt när du återgick till arbetet?

Arbetar du på samma plats idag?

Skulle du vilja berätta lite om ditt arbete?

Vad tycker du om dina arbetsuppgifter/åtaganden?

Hur ser din arbetsbelastning ut i förhållande till din kapacitet?

Finns det något som försvårar arbetet för dig? (t.ex. kroppsliga besvär, minnesproblem, trötthet, tillgänglighet).

Skulle du säga att arbetet är viktigt eller meningsfullt i ditt liv?

*Hälsa (problem kopplade till din stroke)*

Upplever du några problem med din hälsa, nu efter stroke? Det kan vara något fysiskt eller om du mår dåligt psykiskt.

Går du på någon slags rehabilitering?

Tränar du något? Är det något som du skulle vilja göra?

Tycker du att du har fått den hjälp du behöver från sjukhus/vårdcentral när det gäller hur du mår?

Påverkar din hälsa förmågan att känna glädje i livet? (både fysisk och psykisk hälsa)

Känner du att du ibland blir begränsad av din hälsa när du ska företa dig något? (både fysisk och psykisk hälsa)

Har du några särskilda knep eller strategier för att ändå kunna göra det som du önskar? (för att övervinna kroppsliga eller mentala begränsningar)

Har du tillgång till stöd och hjälp från någon i din närhet när det gäller hur du mår? (både fysisk och psykisk hälsa)

*Fritidsintressen (Fritid och kultur)*

Brukar du göra något särskilt som du tycker om på fritiden? (kan du berätta lite om det)

Har du möjlighet att utföra aktiviteter (t.ex. fritidsaktiviteter eller kulturaktiviteter) som du själv tycker om i den mån som du önskar? (om nej)

Vad skulle du säga att det är som hindrar dig från att utföra aktiviteter som du tycker om?

*Tänker du på något sätt annorlunda om ditt liv nu efter stroke?*

Följdfrågor (olika)

*Har det förändrats över tid – d.v.s. hur du tänker kring din delaktighet efter stroke?*

Följdfrågor (olika)

**Be deltagaren definiera: Vad betyder delaktighet för dig? Kan du beskriva en situation i vardagen då du känner dig delaktig?**
